# Interrelationships in the Variability of Root Canal Anatomy among the Permanent Teeth: A Full-Mouth Approach by Cone-Beam CT

**DOI:** 10.1371/journal.pone.0165329

**Published:** 2016-10-20

**Authors:** Paul Monsarrat, Bertrand Arcaute, Ove A. Peters, Elisabeth Maury, Norbert Telmon, Marie Georgelin-Gurgel, Delphine Maret

**Affiliations:** 1 Department of Anatomical Sciences and Radiology, Dental Faculty, Paul Sabatier University, Toulouse University Hospital, Toulouse, France; 2 STROMALab, Université de Toulouse, CNRS ERL 5311, EFS, INP-ENVT, Inserm U1031, UPS, Toulouse, France; 3 Department of Department of Conservative Dentistry and Endodontics, Dental Faculty, Paul Sabatier University, Toulouse University Hospital, Toulouse, France; 4 AMIS Laboratory - Laboratoire Anthropologie Moléculaire et Imagerie de Synthèse, Université de Toulouse, UMR 5288 CNRS, UPS, Toulouse, France; 5 Department of Endodontics, Arthur A. Dugoni School of Dentistry University of the Pacific, San Francisco, California, United States of America; 6 Service de Médecine Légale, Centre Hospitalier Universitaire Rangueil, avenue du Professeur Jean Poulhès, 31059, Toulouse Cedex 9, France; Medical University of South Carolina, UNITED STATES

## Abstract

**Objectives:**

In endodontic practice, clinicians should be aware of possible root canal anatomic variations. The aim of this study was to assess using CBCT acquisitions regarding whether one root canal anatomy of a tooth is associated with a specific anatomy of another tooth.

**Methods:**

A total of 106 CBCT acquisitions were obtained using a CBCT scanner with 200μm voxel size. Numbers of roots and canals of the entire dentition were described. Bivariate analyses and logistic regressions were conducted to explore root canal anatomy on one tooth according to age, gender, jaw, side and the others teeth. Multiple correspondence analysis (MCA) was performed to correlate the different numbers of canals profiles.

**Results:**

A total of 2424 teeth were analyzed. Independently from the other variables, the presence of an additional root canal on a mandibular incisor increases the risk of having an additional root canal on a mandibular premolar (OR _[95%]_ 3.7 [1.0;13.2]). The mandibular molar variability increases in women compared to men (OR _[95%]_ 0.4 [0.1; 0.9]). MCA showed correspondence between 2-canals maxillary incisor and canines and 5-canals maxillary molars, and some correlation between additional canal on maxillary and mandibular premolars.

**Conclusions:**

Although CBCT examinations are conducted in the first intention of making a diagnosis or prognostic evaluation, medium FOV acquisitions could be used as an initial database thus furnishing preliminary evaluations and information. In endodontic practice, clinicians should be aware of possible root canal anatomic variations. The visualization of all canals is considered essential in endodontic therapy. The use of multi-correspondence analysis for statistics in endodontic research is a new approach as a prognostic tool.

## Introduction

Root canal systems have been described as complex anatomical structures with significant implication on root canal preparation. In the literature, various factors such as genetics and ethnic differences, have been reported to influence root canal anatomy [1,2]. Visualization of root canal anatomy is, therefore, of major interest in the practice of dentistry and considered essential in endodontic therapy. Successful root canal treatment is dependent on a detailed understanding of the morphology of the root canal system. Adequate debridement, shaping, and complete obturation in three dimensions are based on knowledge of normal anatomy and variations from the norm. Clinicians should be aware of common root canal configurations and possible anatomic variations [2,3].

Several studies have investigated root canal anatomy with different *ex vivo* methods such as root sectioning, electron microscopy, staining and clearing techniques and micro-computed tomography [4]. Nevertheless, these techniques may lead to a selection bias with a higher proportion of sound teeth or undamaged during extraction [4], and cannot be applied in clinical practice, which generally relies on the use periapical radiographs and more recently on the operating microscope [5].

Conventional radiography yields limited information due to the projection in two dimensions (2D) of a 3-dimensional (3D) anatomical structure, which can lead to failure to recognize a root canal because another structure is superimposed upon it [6]. Cone-beam computed tomography (CBCT) is a considerable technological advancement, providing a three-dimensional view with no overlapping of complex anatomic structures. CBCT scanning can help clinicians view morphological features from a 3-dimensional perspective and to provide the researchers opportunities to study root canal anatomy nondestructively. In endodontic practice, CBCT is a diagnostic tool offering a better understanding of root canal anatomy in axial, sagittal, and coronal sections [7]. Clinical applications differ according to the size of the field of view (FOV) of the CBCT unit.

Considering that the incidence of missed roots or canals in teeth that needed retreatment may be as high as 42% [8], the visualization of all canals is essential. Especially, multi-rooted teeth are described as a complex anatomical structure. In general, maxillary first molars present with 3 roots and 4 canals [9,10]. In the mesiobuccal (MB) root, the incidence of a second canal (MB2) is over 50%, according to the literature [1,6,7,10–12]. However, variations of root canal anatomy concern all teeth. A clinical question is then the capacity to predict the complexity of root canal morphology.

Therefore, the aim of this pilot study was to assess using CBCT acquisitions regarding whether one root canal anatomy of a tooth is associated with a specific anatomy of another tooth.

## Materials and Methods

### Sample

We examined 106 CBCT acquisitions obtained from 2012 to 2013 in a private practice of Oral and Maxillo-Facial Radiology (E. Coudrais EC) in Toulouse, France; all scans were de-identified before being transmitted for analysis (EC). These acquisitions were randomly selected. Authors were not the treating dentists of these patients. Exclusion criteria were patients who were minors, edentulous patients and presence of metallic artifacts. In accordance with ethical and local radiation protection guidelines and the local board, no CBCT examinations were prescribed especially for the study; they were part of routine care. According to French law, the results of medical imaging examinations may be used retrospectively without the patient’s consent when these examinations have been carried out for clinical purposes and when they have been recorded anonymously (article 40–1, law 94–548 of 1 July 1994)[13].

### CBCT scans

The CBCT images were obtained using a CBCT scanner (CS 9500 3D^®^, Carestream, Marne-la-Vallée, France) with tube voltage of 90kV and tube current of 10mA. The voxel size was 200μm and the FOV was 90 x 150mm. The exposure time was 10.8s with a dose—area product of 605 mGy.cm^2^. The scans were acquired by an experienced radiologist (EC) according to the manufacturer’s recommended protocol with the minimum exposure necessary for adequate image quality.

### Image evaluation and study variables

CBCT scans were analyzed using CS Dental Imaging Software 3D Module v3.2.9 (Carestream, Marne-la-Vallée, France). Two dental practitioners independently (B. Arcaute BA and E. Maury EM) evaluated the images twice at an interval of 2 weeks. A preview of the three planes was conducted and following by a visualization of all slices in the axial plan (coronal to apical and apical to coronal directions). The following variables were recorded: age, sex, type of tooth and number of teeth, jaw (maxillary/mandibular) or side (left/right). For each tooth, the number of roots and canals were noted. When reading the scans, third molars and root fragments were excluded. Have also not been considered teeth with orthodontic appliances, stirred images or presence of metallic artifacts preventing the adequate visualization of endodontic structures.

### Statistical analysis

Statistical analysis was performed using the STATA 13.1 software (StataCorp, TX, USA). Inter and intra-examiner agreement was evaluated by the Kappa test. A random sample of 30 CBCT examinations was re-examined one week after the initial examination to test intra-examiner agreement with the Kappa test. The Kappa test was also used to assess inter-examiner agreement, again using a random sample of 30 CBCT examinations. The scans were read by 2 trained observers (BA and EM). The data were analyzed using descriptive statistics. A Stuart-Maxwell test was performed to compare left/right pairs of teeth (paired samples). When a 2x2 comparison was performed, this test acted as the McNemar test. The level of significance was set at 5% (p<0.05).

The effects of age, gender, jaw (maxillary/mandibular) or side (left/right) on the number of roots and canals were analyzed. To achieve this goal, teeth were grouped (maxillary incisors or canines, mandibular incisors or canines, maxillary premolars, mandibular premolars, maxillary molars, mandibular molars); for each patient, a tooth group was considered with a difference compared to the reference group if at least one tooth of the group had a difference from the average dental anatomy.

Non-parametric and unmatched test (Mann-Whitney) was used to determine the effect of age, non-parametric and matched test (Wilcoxon) for the effect of jaw and side, and chi square test for the effect of gender, on variability. The dependent variable of a root anatomical variation for a specific tooth group was analyzed using a logistic regression model, taking into account the other tooth groups, age and gender. Multiple correspondence analysis (MCA) was performed to correlate different numbers of canals profiles.

## Results

### Study sample

From the initial sample of 106 CBCT scans, 102 scans were selected. Four scans were excluded because of presence of a metallic artifact. Patient age ranged from 20 to 88 years, with a mean age of 46.8 years. The study sample included 53 women and 49 men. The inter-examiner agreement of 30 CBCT scans was very high with a Kappa coefficient of 0.86 for root numbers and 0.82 for canal numbers. The intra-examiner agreement of 30 CBCT scans was also very high with a Kappa coefficient of 0.97 for both root and canal numbers.

### Descriptive data

A total of 2424 teeth were analyzed (1199 maxillary teeth and 1225 mandibular teeth). There was no significant difference between men and women regarding their age or the number of teeth in each dental arch (Table 1).

**Table 1:** Description of study population.

	Number of patients	Number of teeth	Age
	N (%)	Mean ± SD	Mean ± SD (min–max)
**Total**	102	25.2 ± 4.3	46.8 ± 15.9 (20–88)
Men	49 (48%)	25.5 ± 4.6	45.5 ± 15.9 (20–81)
Women	53 (52%)	24.9 ± 4.0	48.0 ± 15.9 (23–88)

We detected no significant different between men and women regarding the age or the number of teeth on dental arch.

### Number of roots and canals

Table 2 summarizes the details regarding the number of teeth, canals and roots.

**Table 2:** Details about the number of canals and roots observed in the medium field of view cone-beam CT acquisitions.

ISO	UNS	n	1 canal 1root	2 canals 1root	2 canals 2 roots	3 canals 1root	3 canals 2 roots	3 canals 3 roots	3 canals 4 roots	4 canals 2 roots	4 canals 3 roots	4 canals 4 roots	5 canals 3 roots	5 canals 4 roots
**11**	**8**	97	*96 (99%)*	-	1 (1%)	-	-	-	-	-	-	-	-	-
**21**	**9**	95	*94 (99%)*	-	1 (1%)	-	-	-	-	-	-	-	-	-
**31**	**24**	96	*84 (88%)*	11 (11%)	1 (1%)	-	-	-	-	-	-	-	-	-
**41**	**25**	96	*83 (86%)*	12 (13%)	1 (1%)	-	-	-	-	-	-	-	-	-
**12**	**7**	96	*95 (99%)*	-	1 (1%)	-	-	-	-	-	-	-	-	-
**22**	**10**	95	*94 (99%)*	-	1 (1%)	-	-	-	-	-	-	-	-	-
**32**	**23**	100	*87 (87%)*	12 (12%)	1 (1%)	-	-	-	-	-	-	-	-	-
**42**	**26**	99	*85 (86%)*	13 (13%)	1 (1%)	-	-	-	-	-	-	-	-	-
**13**	**6**	94	*93 (99%)*	-	1 (1%)	-	-	-	-	-	-	-	-	-
**23**	**11**	94	*93 (99%)*	-	1 (1%)	-	-	-	-	-	-	-	-	-
**33**	**22**	101	*96 (95%)*	3 (3%)	2 (2%)	-	-	-	-	-	-	-	-	-
**43**	**27**	101	*95 (94%)*	1 (1%)	5 (5%)	-	-	-	-	-	-	-	-	-
**14**	**5**	81	9 (11%)	2 (3%)	*66 (81%)*	-	1 (1%)	3 (4%)	-	-	-	-	-	-
**24**	**12**	84	14 (17%)	1 (1%)	*64 (84%)*	-	1 (1%)	4 (5%)	-	-	-	-	-	-
**34**	**21**	95	*82 (87%)*	7 (7%)	6 (6%)	-	-	-	-	-	-	-	-	-
**44**	**28**	92	*81 (88%)*	6 (7%)	5 (5%)	-	-	-	-	-	-	-	-	-
**15**	**4**	73	*49 (67%)*	4 (6%)	19 (26%)	-	-	1 (1%)	-	-	-	-	-	-
**25**	**13**	74	*49 (66%)*	3 (4%)	20 (27%)	-	-	2 (3%)	-	-	-	-	-	-
**35**	**20**	79	*75 (95%)*	3 (4%)	1 (1%)	-	-	-	-	-	-	-	-	-
**45**	**29**	80	*78 (98%)*	1 (1%)	1 (1%)	-	-	-	-	-	-	-	-	-
**16**	**3**	78	-	-	1 (1%)	-	1 (1%)	37 (48%)	-	1 (1%)	*37 (48%)*	-	-	1 (1%)
**26**	**14**	71	-	-	-	-	-	30 (42%)	-	-	*40 (56%)*	-	-	1 (2%)
**36**	**19**	67	-	2 (3%)	-	-	*42 (62%)*	1 (2%)	1 (2%)	19 (28%)	2 (3%)	-	-	-
**46**	**30**	63	-	-	-	-	*46 (73%)*	3 (5%)	-	10 (16%)	4 (6%)	-	-	-
**17**	**2**	84	1 (1%)	-	-	3 (4%)	3 (4%)	*55 (65%)*	-	1 (1%)	19 (23%)	2 (2%)	-	-
**27**	**15**	83	1 (1%)	-	-	5 (6%)	2 (3%)	*48 (58%)*	-	-	25 (30%)	1 (1%)	-	1 (1%)
**37**	**18**	75	-	-	1 (1%)	-	*66 (88%)*	5 (7%)	-	1 (1%)	2 (3%)	-	-	-
**47**	**31**	81	2 (2%)	-	3 (4%)	-	*62 (77%)*	8 (10%)	-	3 (4%)	2 (2%)	-	1 (1%)	-

The group in underline italics represented the group taken as a reference. We used the ISO and Universal Numbering System (UNS).

#### Incisors and canines

**Fig 1:**
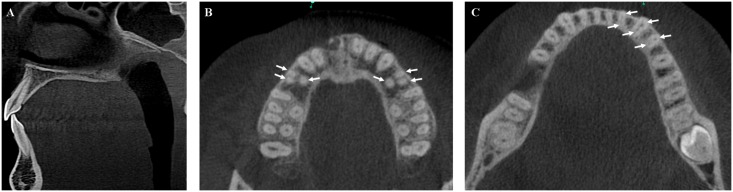
CBCT acquisitions to illustrate the presence of additional canals. (A) Sagittal CBCT image of a mandibular central incisor with one root and two canals. (B) Presence of an additional canal bilaterally in first maxillary premolars (white arrows). (C) Additional canal (white arrows) concomitantly on mandibular incisors, canines and first mandibular premolars, together with no variability on mandibular molars (3 canals, 2 roots).

Morphological examination of the maxillary and mandibular incisor-canine group showed that these teeth generally had one root for one canal (Fig 1). Anatomical variations were more frequent in the mandibular central and lateral incisors, where 2 canals for one root were identified in 11% of left central incisors, 13% of right central incisors, 12% of left lateral incisors, and 13% of right lateral incisors (Fig 1).

#### Premolars

The first maxillary premolar generally had 2 canals for 2 roots but 3 canals for 3 roots were also found (Table 2). The second maxillary premolar generally showed 1 canal for 1 root. In the mandible, the majority of premolars had a single root with a single canal. However, the first premolar showed more variations, with 2 canals for one root or 2 canals for 2 roots.

#### Molars

The most common morphology for maxillary molars was 3 roots for both first and second molars (Table 2). Maxillary first molars presented with 4 canals in about half of the cases, while second molars in general had 3 roots and 3 canals; however, 4-canal, 3-root and 3-canal as well as 1-root morphologies were also present. The mandibular first molar generally had 3 canals and 2 roots or 4 canals and 2 roots (Table 2). The second molar more generally had 3 canals and 2 roots (Table 2). However, the presence of 3 canals and 3 roots was less frequent (left 7%, right 10%).

Table 3 presents the proportion of tooth groups, which have at least one difference from the typical dental anatomy presented in Table 2. For example, a difference compared to reference group in “the mandibular incisors or canines” was attributed to patient if all these teeth didn’t have 1 canal 1 root (Table 2); these differences constituted the variability group. The greatest variability was demonstrated for maxillary molars; 80% of these teeth didn’t have 4 canals 3 roots for first maxillary molars and 3 canals 3 roots for second maxillary molars (Table 3). Maxillary premolars and mandibular molars had 54% and 41% variability, respectively. The lower variability was observed for mandibular and maxillary incisors or canines, and mandibular premolars groups (Table 3). Both the number of canals and the number of roots for these teeth were lower in the reference group compared to the variability group (S1 Table).

**Table 3:** Proportion of tooth groups which have at least a difference from the average dental anatomy presented in Table 2.

	Variability group (difference compared to reference group)	Reference group
**Maxillary incisors or canines**	1 (1%)	100 (99%)
**Mandibular incisors or canines**	25 (25%)	77 (75%)
**Maxillary premolars**	53 (54%)	45 (46%)
*Maxillary first premolar*	*24 (26%)*	*68 (74%)*
*Maxillary second premolar*	*33 (39%)*	*52 (61%)*
**Mandibular premolars**	18 (18%)	83 (82%)
**Maxillary molars**	77 (80%)	19 (20%)
*Maxillary first molar*	*48 (55%)*	*40 (45%)*
*Maxillary second molar*	*44 (48%)*	*48 (52%)*
**Mandibular molars**	39 (41%)	57 (59%)

### Bivariate analysis between each predictive factor

#### Age

An age difference was observed for mandibular premolars: reference group was significantly more aged (48.4 ± 1.7 versus 39.5 ± 3.2 years, p = 0.04).

#### Gender

The proportion of women was lower in the variability group compared to the reference group for maxillary premolars (43% versus 62%, p = 0.05), whereas higher for mandibular molars (62% versus 42%, p = 0.05). Globally, both the mean number of roots and canals were lower for maxillary premolars and greater for mandibular molars, when women were compared to men (S2 Table).

#### Jaw factor

An increased variability was observed for mandibular incisors and canines compared to maxillary (p<0.001). The number of canals for incisors, and the number of roots and canals for canines were increased (Table 2). An increased variability was detected for maxillary premolars compared to mandibular (p<0.001); the proportion of second premolars with 2 canals/2 roots (26–27%) was much higher in maxillary compared to mandibular (1%) (Table 2). Except for 5 and 12, premolar variability involved more canals and more roots. For molars, the greatest variability concerned maxillary (p<0.001); it was found that about half first maxillary molars were concerned by 3 canals/4 roots and half by 3 canals/3 roots, whereas two third mandibular molars were concerned by 3 canals/2 roots and one third by 4 canals/2 roots (Table 2). Finally, we observed that mandibular molar variability implied more canals and more roots, whereas it was not the case for maxillary: first molar variability implied less canals and less roots, second molar variability implied more canals and less roots (S1 Table).

#### Side factor

The Stuart-Maxwell test did not reveal any significant difference between the right and left side in the present study. However, there appeared to be a trend to more first mandibular molars with 2 roots and 4 canals on the right side (p = 0.06).

### Multifactorial analysis of presence of additional root canals in specific tooth groups (Table 4)

**Table 4:** Results from logistic regression analysis (90 observations).

	Dependent variable					
OR_[95%]_	Maxillary incisors/canines	Mandibular incisors/canines	Maxillary premolars	Mandibular premolars	Maxillary molars	Mandibular molars
**Explanatory variables**						
Maxillary incisors/canines	-	-	-	-	-	-
Mandibular incisors/canines	-	-	0.7 [0.2;1.9]	*3*.*7 [1*.*1;13*.*2]*	1.8 [0.4;7.8]	1.7 [0.5;4.8]
Maxillary premolars	-	0.6 [0.2;1.8]	-	2.2 [0.6;7.8]	0.5 [0.1;1.5]	1.4 [0.5;3.6]
Mandibular premolars	-	*4*.*3 [1*.*2;15*.*4]*	2 [0.6;6.8]	-	4.3 [0.5;38.8]	*0*.*2 [0*.*1;0*.*9]*
Maxillary molars	-	2 [0.5;8.7]	0.5 [0.1;1.5]	4 [0.5;42.9]	-	0.7 [0.2;2.1]
Mandibular molars	-	1.7 [0.6;4.9]	1.4 [0.5;3.6]	0.3 [0.1;1.1]	0.7 [0.2;2.2]	-
Age	-	*1*.*1 [1;1*.*1]*	1 [0.9;1.1]	*0*.*9 [0*.*8;0*.*9]*	1 [0.9;1.1]	*0*.*9 [0*.*8;0*.*9]*
Sex	-	2.6 [0.8;7.6]	2.2 [0.8;5.4]	0.6 [0.2;2.3]	0.7 [0.2;2.1]	*0*.*4 [0*.*1;0*.*9]*

The odds ratio (OR = e^β^) for each covariate in underline italics represented significant results at 95% confidence interval (OR_[95%]_).

Taking into account the other tooth groups, age and gender, having variability on a mandibular premolar increased 4 times the risk to have variability on mandibular incisors or canines (OR _[95%]_ 4.3[1.2; 15.4]). Conversely, having variability on mandibular incisors or canines increased 4 times the risk to have variability on mandibular premolars (OR _[95%]_ 3.7 [1.0;13.2]). Having variability on mandibular premolars decreased the risk to discover variability on mandibular molars (OR _[95%]_ 0.2 [0.1;0.9]). Finally, being a male decreased about twice the risk to have a variability on mandibular molars compared to being a female (OR _[95%]_ 0.4 [0.1;0.9]). While on some occasions significant, the factor “age” overall contributed minimally to models.

**Fig 2:**
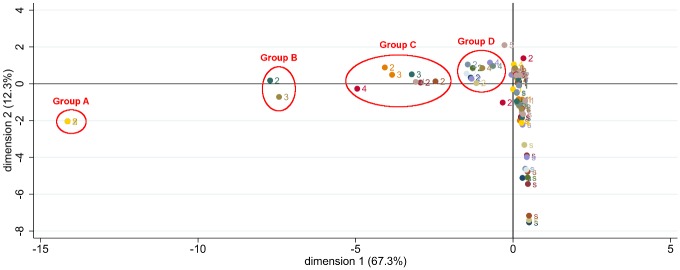
Multiple correspondence analysis MCA. When the tooth was absent, the number of canals was considered as a new category “s” to be included in the 102 observations analysis of the MCA.

The first dimension of MCA always explained at least 67% of the principal inertia, while the second dimension explained only 12%, and the following dimensions less than 3% (Fig 2). This tended to validate the one-dimensionality. Group A showed correspondence between 2-canals maxillary incisor and canines and 5-canals maxillary molars. Group B and C revealed some correspondence between additional canal on maxillary and mandibular premolars. Group C and D identified some correspondence between additional canal on mandibular incisors or canines and molars (Table 5).

**Table 5:** Details about each group according to MCA analysis (ISO system).

**Group A**	11: 2-canals
	12: 2-canals
	13: 2-canals
	21: 2-canals
	22: 2-canals
	23: 2-canals
	15: 3-canals
	16: 5-canals
	26: 5-canals
	27: 5-canals
**Group B**	25: 3-canals
	45: 2-canals
**Group C**	14: 3-canals
	24: 3-canals
	33: 2-canals
	43: 2-canals
	35: 2-canals
	37: 4-canals
	47: 4-canals
**Group D**	17: 4-canals
	31: 2-canals
	32: 2-canals
	34: 2-canals
	41: 2-canals
	42: 2-canals
	44: 2-canals
	36: 4-canals
	46: 4-canals

## Discussion

The visualization of all canals is considered essential in endodontic therapy. CBCT images are known to provide three-dimensional information about the presence of additional canals (Fig 1). The use of CBCT imaging with a resolution of 200μm is a limitation of the present study. For example the coarser resolution creates uncertainly when describing canal anatomy according to Vertucci’s classification [3,14]. Also, studies using an operating microscope, sectioning methodology or clearing technique revealed a higher canal detection rate than CBCT examinations [6]. In fact, due to the discrete grid of a CBCT reconstruction matrix, any canal with a maximum cross-sectional dimension of less than 400μm could, in a worst-case scenario, escaped detection in this data set.

Using CBCT acquisitions of 90x150mm FOV, entire upper and lower dental arches can be visualized. Consequently, intra-individual dental variability, the relationship between the additional canals found among the different groups of teeth, may be analyzed.

Consequently, this study will bring elements for the clinician, allowing it to anticipate the degree of technical difficulty and any radiological investigation based on anatomical variations already discovered on other teeth. For example, discovering two canals in a mandibular incisor multiplied by 4 the risk to discover two canals in a mandibular premolar, and conversely.

In the maxillary incisor-canine group, only slightly over 1% of teeth had more than one root or canal. These results are consistent with the literature [14]. Observation of mandibular incisor-canine teeth highlighted the variable canal anatomy of the incisors. They had 2 canals in one root in 11% to 13%. The presence of 2 canals was found in previous studies but in variable proportions ranging from 0.3% to 45.3% depending on the techniques used [15,16]. The maxillary first premolars showed a large incidence of teeth with 2 roots and 2 canals, observed in between 81% to 84%. These results were consistent with previous studies [17]. A third root and a third canal were present in our study in less than 5% of cases. The maxillary second premolars frequently had a single root and a single canal per root. Others studies differed, with either a higher [18] or a lower incidence of 2 canals per root [19]. The presence of 3 canals and 3 roots was rare in our study, at less than 3%. Mandibular premolars frequently had a single root in between 94% to 99% of the cases, which is similar to findings by Cleghorn *et al*. [20,21]. In our study, we mainly found a single canal between 87% to 98%. However, the mandibular first premolar had 2 canals in 12% (right) to 13% of cases (left). The prevalence appears to be lower than that observed by Cleghorn *et al*. [20], who found that 24.2% of first premolars had two or more canals.

Several authors have studied maxillary molar anatomy. The presence of 3 separate roots was the most common configuration of the maxillary first and second molars [3]. In addition, the first molar had 3 canals for 3 roots in 42% (left) to 48% (right) and 4 canals for 3 roots in 48% (right) to 56% (left). The results showed that around 5% of maxillary first molars did not have three roots. These results were consistent with previous studies of Chinese, Korean, Brazilian and Indian populations [7,10,12]. However, other studies of Burmese or Thai populations [22,23] showed 3 separate roots for all maxillary first molars. These differences suggested the potential role of ethnicity [10]. Nevertheless, the relationship of ethnic background to anatomic variants was not explored in current sample; it is a potential limitation for the current study since majority of the population studied is expected to be Caucasian.

The morphology most commonly observed for maxillary second molars was 3 roots, with one canal in each root (left 58%, right 65%). The presence of a fourth canal was recorded in 23% (right) to 30% (left), which was similar to previous studies [10,22,23]. Mandibular molars have also been studied in the literature [2,10,12,24]. The mandibular first molar had two roots in 89% (right) to 91% (left) of cases. The most common finding was 3 canals and 2 roots (62% on the left and 73% on the right), according to previous studies [10,25]. The role of ethnicity in the morphology of the mandibular first molar has been stressed in studies, with a higher prevalence of teeth with 3 roots in East Asian populations [25]. With regard to the mandibular second molar, the most frequent morphology was 2 separate roots and 3 canals. Although the literature concluded that this was the most common configuration, significant differences were observed between different populations [5,10,24].

The second part of our study objectives was to conduct logistic regression analyses to obtain the risk to discover additional canal on a group of teeth when a variability was found on another group of teeth. To the best of our knowledge, few investigations have looked for left-right symmetry in pairs of opposite teeth, and when such studies have been performed, they have only considered one type of tooth and not the entire dentition [26,27]. In our study, the Stuart-Maxwell test did reveal a trend of a difference between the left and right mandibular molars (p = 0.06). First left mandibular molars in this study have a tendency to more canals and less roots compared to the first right mandibular molars. Such results were consistent with Kim *et al*. which found side asymmetry, with a right-sided predominance for extra distal roots and a left-sided predominance for extra distal canals [25].

Root canal anatomy studies with CBCT have described a single type of tooth [18,25] or a group of teeth [28], but not all teeth present on the maxillary and mandibular arches. These studies often used a small FOV with a small voxel size (e.g 76μm), which makes it possible to see the root canal anatomy in detail. Such acquisitions were mainly performed during clinical endodontic practice, more precisely in the presence of clinical signs and symptoms (2015 AAE and AAOMR Joint Position Statement). Accordingly, there was an increased risk to discover more complex root canal anatomies (risk of teeth selection bias). However, studies to predict additional canals need the visualization of all the teeth in one examination (*i*.*e*. with medium FOV). Independently from the other variables (i.e. age, gender, other teeth), the presence of an additional root canal on a mandibular incisor increases the risk of having an additional root canal on a mandibular premolar. The variability on mandibular premolars and molars appears to decrease with age. This can be explained by the root canal mineralization and highlights the difficulty to visualize small additional canals. Bivariate and multivariate analyses identified some association between gender and the presence of an additional canal. The increased variability of second maxillary premolars in men compared to women was reported in some studies [29,30]. Mandibular molar root variability is more contrasted: many studies have found male predominance [31], but some studies have also reported an increased anatomical variation in women compared to men, as we pointed-out [32].

Even if a probabilistic model has been reported in endodontic research to obtain probability of an additional root in mandibular molars, the use of MCA in statistical analyses is a new and original approach. This correspondence analysis model enabled us to form four groups with different number of canals.

## Conclusions

The visualization of all canals is considered relevant in endodontic therapy. Can the presence of an additional canal on one tooth predict the chance to discover an additional canal on another tooth in the same patient? Although CBCT examinations are conducted in the first intention of making a diagnosis or prognostic evaluation, medium FOV acquisitions could be used as an initial database thus furnishing preliminary evaluations and information. Multi-institutional studies may be undertaken to collect more CBCT images and perform additional statistical analyses.

## Supporting Information

S1 Table Considering the presence or absence of variability in each tooth group, the mean number ± standard deviation of canals and roots for each tooth were provided. (DOCX)

S2 Table For each tooth, the mean number ± standard deviation of canals and roots was presented, for men and women. (DOCX)
