# Supplementary material for: Interrelationships in the Variability of Root Canal Anatomy among the Permanent Teeth: A Full-Mouth Approach by Cone-Beam CT
Source: PLoS One. 2016 Oct 20;11(10):e0165329. doi: 10.1371/journal.pone.0165329 (PMC5072733; doi:10.1371/journal.pone.0165329)
Supplement: S1 Table — (DOCX) [file pone.0165329.s001.docx]

**S1 Table.**

|  | **Tooth** | | **Reference group**  **(no variability)** | | **Difference compared to reference group** - Mean ± SD  **(variability)** | | |
| --- | --- | --- | --- | --- | --- | --- | --- |
| **Variability in tooth group** | **ISO** | **UNS** | **Canals** | **Roots** | **Variability (n, %)** | **Number of canals** | **Number of roots** |
| **Maxillary incisors or canines** | **11** | 8 | 1 | 1 | 1 (1%) | 2 | 2 |
|  | **12** | 7 | 1 | 1 | 1 (1%) | 2 | 2 |
|  | **13** | 6 | 1 | 1 | 1 (1%) | 2 | 2 |
|  | **21** | 9 | 1 | 1 | 1 (1%) | 2 | 2 |
|  | **22** | 10 | 1 | 1 | 1 (1%) | 2 | 2 |
|  | **23** | 11 | 1 | 1 | 1 (1%) | 2 | 2 |
| **Mandibular incisors or canines** | **31** | 24 | 1 | 1 | 12 (13%) | 1.5 ± 0.5 | 1 ± 0.2 |
|  | **32** | 23 | 1 | 1 | 13 (13%) | 1.5 ± 0.5 | 1 ± 0.2 |
|  | **33** | 22 | 1 | 1 | 5 (5%) | 1.2 ± 0.4 | 1.1 ± 0.3 |
|  | **41** | 25 | 1 | 1 | 13 (14%) | 1.5 ± 0.5 | 1 ± 0.2 |
|  | **42** | 26 | 1 | 1 | 14 (14%) | 1.5 ± 0.5 | 1.1 ± 0.2 |
|  | **43** | 27 | 1 | 1 | 6 (6%) | 1.2 ± 0.4 | 1.2 ± 0.4 |
| **Maxillary premolars** | **14** | 5 | 2 | 2 | 15 (19%) | 1.8 ± 0.5 | 1.8 ± 0.5 |
|  | **15** | 4 | 1 | 1 | 24 (33%) | 1.6 ± 0.5 | 1.5 ± 0.5 |
|  | **24** | 12 | 2 | 2 | 20 (24%) | 1.8 ± 0.6 | 1.7 ± 0.6 |
|  | **25** | 13 | 1 | 1 | 25 (34%) | 1.6 ± 0.6 | 1.5 ± 0.6 |
| **Maxillary molars** | **16** | 3 | 4 | 3 | 41 (53%) | 3.4 ± 0.5 | 2.9 ± 0.2 |
|  | **17** | 2 | 3 | 3 | 29 (35%) | 3.3 ± 0.5 | 2.8 ± 0.5 |
|  | **26** | 14 | 4 | 3 | 31 (44%) | 3.5 ± 0.5 | 3 ± 0.1 |
|  | **27** | 15 | 3 | 3 | 35 (42%) | 3.4 ± 0.6 | 2.8 ± 0.6 |
| **Mandibular premolars** | **34** | 21 | 1 | 1 | 13 (14%) | 1.7 ± 0.5 | 1.3 ± 0.5 |
|  | **35** | 20 | 1 | 1 | 4 (5%) | 1.3 ± 0.5 | 1.1 ± 0.3 |
|  | **44** | 28 | 1 | 1 | 11 (12%) | 1.6 ± 0.5 | 1.3 ± 0.5 |
|  | **45** | 29 | 1 | 1 | 2 (3%) | 1.1 ± 0.4 | 1.1 ± 0.3 |
| **Mandibular molars** | **36** | 19 | 3 | 2 | 25 (37%) | 3.6 ± 0.6 | 2.1 ± 0.5 |
|  | **37** | 18 | 3 | 2 | 9 (12%) | 3.1 ± 0.3 | 2.2 ± 0.4 |
|  | **46** | 30 | 3 | 2 | 17 (27%) | 3.5 ± 0.5 | 2.3 ± 0.4 |
|  | **47** | 31 | 3 | 2 | 19 (23%) | 3 ± 0.7 | 2.2 ± 0.5 |

Considering the presence or absence of variability in each tooth group, the mean number ± standard deviation of canals and roots for each tooth were provided.
