# Supplementary material for: Interrelationships in the Variability of Root Canal Anatomy among the Permanent Teeth: A Full-Mouth Approach by Cone-Beam CT
Source: PLoS One. 2016 Oct 20;11(10):e0165329. doi: 10.1371/journal.pone.0165329 (PMC5072733; doi:10.1371/journal.pone.0165329)
Supplement: S2 Table — (DOCX) [file pone.0165329.s002.docx]

**S2 Table.**

| **Tooth** | | **Women** (Mean ± SD) | | **Men** (Mean ± SD) | |
| --- | --- | --- | --- | --- | --- |
| **ISO** | **UNS** | **Number of canals** | **Number of roots** | **Number of canals** | **Number of roots** |
| **14** | **5** | 1.9 ± 0.4 | 1.9 ± 0.4 | 1.9 ± 0.4 | 1.9 ± 0.4 |
| **15** | **4** | 1.2 ± 0.4 | 1.2 ± 0.4 | 1.4 ± 0.5 | 1.3 ± 0.5 |
| **24** | **12** | 1.8 ± 0.5 | 1.8 ± 0.4 | 1.9 ± 0.5 | 1.8 ± 0.5 |
| **25** | **13** | 1.2 ± 0.4 | 1.1 ± 0.3 | 1.5 ± 0.6 | 1.5 ± 0.6 |
| **36** | **19** | 3.4 ± 0.5 | 2.1 ± 0.4 | 3.2 ± 0.5 | 2 ± 0.3 |
| **37** | **18** | 3 ± 0.3 | 2.1 ± 0.3 | 3 ± 0.1 | 2.1 ± 0.3 |
| **46** | **30** | 3.3 ± 0.4 | 2.1 ± 0.4 | 3.1 ± 0.4 | 2.1 ± 0.2 |
| **47** | **31** | 2.9 ± 0.7 | 2.1 ± 0.5 | 3.1 ± 0.2 | 2.1 ± 0.2 |

For each tooth, the mean number ± standard deviation of canals and roots was presented, for men and women.
